# Exploring genetic structures and shared sites between alcohol, cheese intake, and inflammatory bowel disease

**DOI:** 10.3389/fnut.2025.1468457

**Published:** 2025-01-23

**Authors:** Zhifang Huang, Weichao Yuan

**Affiliations:** ^1^Department of Anorectal Surgery, Jiangmen Wuyi Hospital of Traditional Chinese Medicine, Jiangmen, China; ^2^Department of Anorectal Surgery, Affiliated Hospital of Jiujiang University, Jiujiang, China

**Keywords:** genetic structures, shared sites, alcohol intake, cheese intake, inflammatory bowel disease

## Abstract

**Background:**

An association has been observed between alcohol and cheese intake and the onset of inflammatory bowel disease (IBD), necessitating further exploration from a genetic structural perspective.

**Methods:**

The present analysis was focused on the intake of alcohol and cheese in conjunction with IBD genome-wide association study (GWAS) data, with the objective of exploring genetic correlations and identifying common loci. Initially, overall genetic correlations were assessed employing two methodologies: linkage disequilibrium score regression (LDSC) and genetic covariance analyzer (GNOVA). Subsequently, local correlations were examined through the SUPERGNOVA method. A genetic overlap analysis between various traits was then conducted based on the statistical theory of conditional/conjunctional false discovery rate (cond/conjFDR). Ultimately, shared loci between the two traits were identified via conjFDR analysis and multi-trait analysis of GWAS (MTAG).

**Results:**

Substantial overall correlations were noted at the genome-wide level between alcohol and cheese intake and both IBD and Crohn’s disease (CD), whereas the association with ulcerative colitis (UC) was of lesser significance. In the local genetic analysis, chromosome 16 emerged as a key region implicated in the relationship between alcohol and cheese intake and IBD (including both CD and UC). The conjFDR analysis confirmed the genetic overlap between the two diseases. Furthermore, both conjFDR and MTAG analyses identified multiple shared genetic loci, with nine genes (*Y_RNA*, *DENND1B*, *GCKR*, *KPNA7*, *CLN3*, *SLC39A8*, *FUT2*, *ERAP2*, and *SMAD3*) being.

**Conclusion:**

The present study provides genetic evidence supporting the comorbidity of alcohol and cheese intake with IBD, offering novel insights into potential strategies for the prevention and treatment of IBD through the modulation of alcohol and cheese consumption.

## 1 Introduction

Inflammatory bowel disease (IBD) is characterized as a non-specific, immune-mediated, chronic, relapsing gastrointestinal disorder, which is further classified into Crohn’s disease (CD) and ulcerative colitis (UC) (1). The global incidence of IBD has been rising, with the prevalence in Western populations projected to reach 1% by 2030, thereby imposing a considerable burden on global health and economies (2, 3). Currently, a comprehensive understanding of the etiology and pathogenesis of IBD remains incomplete. Furthermore, factors such as genetic predisposition, a compromised intestinal mucosal barrier, urbanization, dietary components, and disruptions in gut microbiota and mucosal immunity have been implicated, any of which could potentially trigger the onset of IBD (4). Alcohol and cheese intake, which are common dietary practices in many cultures, have been reported to be significantly linked with the development of IBD (5, 6). Given that observational studies are frequently influenced by confounding factors, a reevaluation of these associations from the standpoint of genetic overlap is intended.

In recent years, genome-wide association studies (GWAS) pertaining to both IBD and dietary habits, such as alcohol and cheese intake, have advanced to a more sophisticated stage, thereby establishing a foundation for investigating the genetic overlap between these traits. Currently, numerous innovative and reliable statistical genetic methods have been developed. LD Score Regression (LDSC) estimates genome-wide genetic correlations using GWAS summary statistics while accounting for linkage disequilibrium (LD) and sample overlap (7). Genetic covariance analyzer (GNOVA) calculates annotation-stratified genetic covariance, providing precise insights into shared genetic components (8). SUPERGNOVA identifies local genetic correlations, addressing challenges such as extensive LD and sample overlap to uncover heterogeneous genetic sharing (9). Conjunctional False Discovery Rate (ConjFDR) detects shared genetic loci by leveraging cross-trait SNP enrichment and maximizing conditional FDR values (10). Lastly, Multi-Trait Analysis of GWAS (MTAG) enhances statistical power to identify trait-specific genetic associations by integrating summary statistics from multiple traits while accounting for genetic correlation and sample overlap (11). Together, these methods provide a comprehensive framework for uncovering shared genetic architectures. These methods, including LDSC, ConjFDR, and MTAG, have demonstrated their utility in identifying shared genetic architectures between schizophrenia and metabolic syndrome (12), uncovering both global and local genetic correlations between inflammatory bowel disease and systemic lupus erythematosus (using GNOVA and SUPERGNOVA) (13), and systematically exploring the shared genetics between Alzheimer’s disease and cardiovascular traits (involving MTAG) (14). The genetic associations between IBD and alcohol and cheese intake remain unclear. To enhance the investigation of the genetic overlap between IBD and alcohol and cheese intake, these methods have been adapted with the aim of providing a more comprehensive understanding of their shared genetic framework.

This study aims to address critical gaps in our understanding of the genetic associations between IBD and dietary factors, specifically alcohol and cheese intake, by focusing on two main objectives: (1) to evaluate the genetic correlation between these traits and (2) to identify shared genetic loci. By employing state-of-the-art statistical methodologies, this research contributes to the field by providing novel insights into the shared genetic architecture between dietary factors and IBD, thereby advancing the current knowledge of their potential interactions. Specifically, we utilize LDSC (7) and GNOVA (8) to assess genome-wide genetic correlations, while SUPERGNOVA (9) is implemented to explore local genetic correlations. For shared loci identification, we leverage cond/conjFDR (10) and MTAG (11), both of which are well-established methods for identifying genetic risk loci across comorbid traits. The findings from this study are expected to offer significant contributions by clarifying the genetic underpinnings linking alcohol and cheese intake to IBD and potentially guiding future research in nutritional genomics and precision medicine.

## 2 Materials and methods

### 2.1 GWAS data

**Figure 1:**
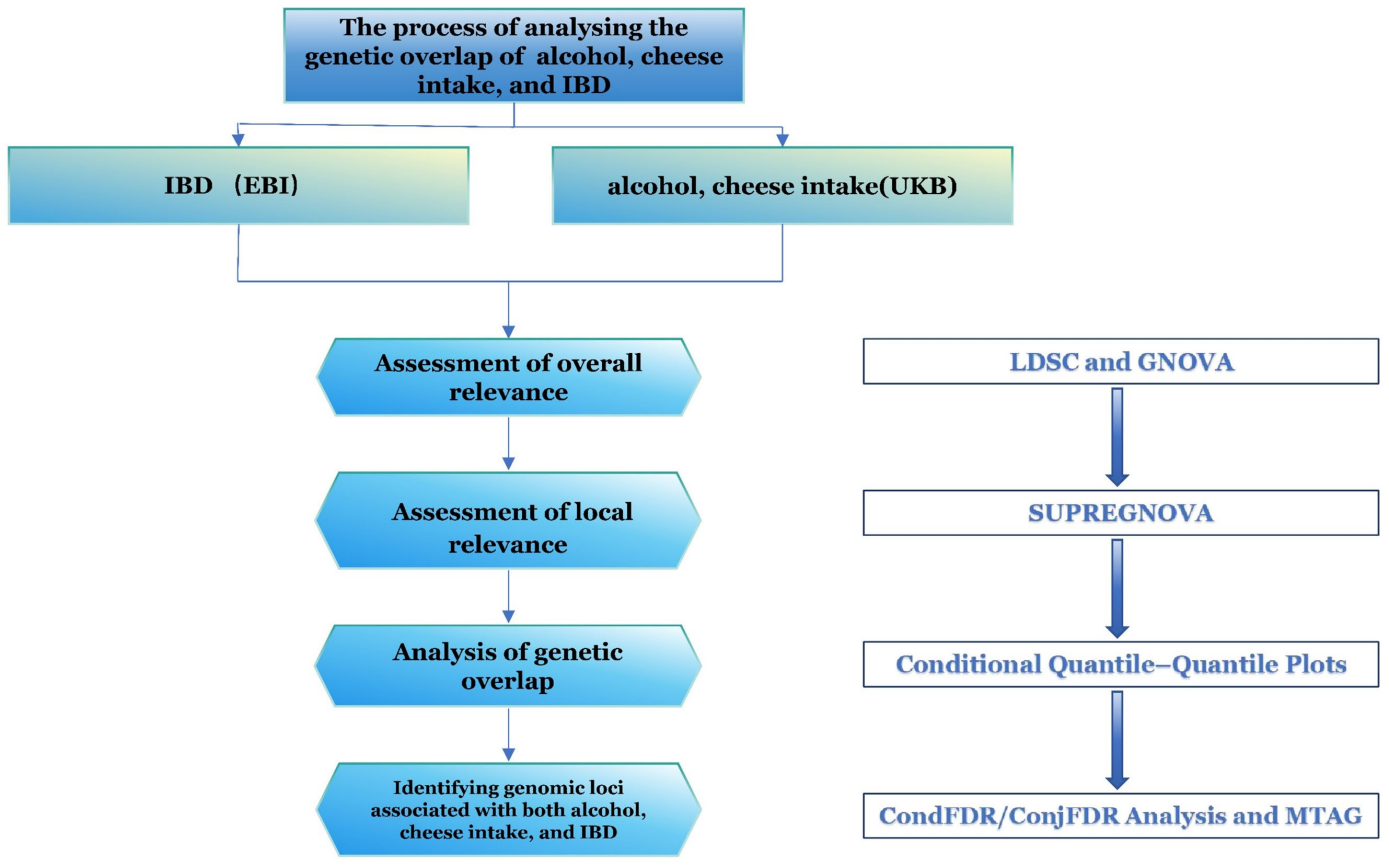
Flowchart of the study. IBD, inflammatory bowel disease; LDSC, linkage disequilibrium score regression; MTAG, multi-trait analysis; TWAS, transcriptome-wide association studies.

The GWAS data for IBD and its subtypes (CD and UC), as provided by de Lange et al. (15), were selected for this study due to their representation of the largest and most recent patient cohort. The GWAS data for alcohol and cheese intake were obtained from the IEU GWAS database^1^, with the corresponding IDs “ukb-b-5779” and “ukb-b-15926,” respectively. The study populations in these datasets were exclusively of European ancestry. For alcohol intake data, the definition includes the overall intake of all forms of alcoholic beverages but does not explicitly distinguish between fermented alcoholic drinks (e.g., wine and beer) and distilled alcoholic beverages (e.g., spirits). While this definition provides a comprehensive measure of overall alcohol intake, it may fail to capture the specific effects of different types of alcoholic beverages on disease associations. In our study design, we acknowledged this limitation and further analyzed it in the discussion section. The flow chart of this study is shown in Figure 1.

### 2.2 Global and local genetic correlation analyses

The LDSC and GNOVA are particularly advantageous for investigating overall genetic correlations. The LDSC calculation process is comprised of two steps: ① transforming all GWAS datasets into the LDSC format through the use of the munge_sumstats.py parameters, and ② estimating the genetic correlation (rg) between two traits by applying the -rg, −ref-ld-chr, and -w-ld-chr parameters. Genetic covariance is assessed by GNOVA through the exploitation of shared genetic variants between the two diseases (8). The genetic correlation is subsequently derived from the genetic covariance and trait heritability. The rg value, a critical metric in both analyses, ranges from −1 to +1. The sign (“±”) indicates the direction of the effect, while the magnitude reflects the strength of the correlation. A Bonferroni correction is applied to adjust the *p*-values.

SUPERGNOVA is utilized to assess the correlation between two traits across various chromosomal locations. The entire genome is partitioned into 2,353 segments, and the similarity between trait pairs, influenced by genetic variations in each region, is determined (9). When the *p*-value of a corresponding segment falls below the Bonferroni-corrected threshold (*p* < 0.05/2,353), the segment is considered statistically significant, indicating a locus with a local genetic association.

### 2.3 Conditional quantile–quantile plots

The conditional quantile-quantile (Q–Q) plot is utilized as a visual tool to illustrate genetic overlap results, highlighting the enrichment of polygenic effects across phenotypes. The Q–Q plot is divided into three *p*-value segments: “*p* < 0.10,” “*p* < 0.01,” and “*p* < 0.001.” If, as the *p*-value decreases, the proportion of SNPs associated with one phenotype (e.g., alcohol intake) progressively shifts leftward relative to another phenotype (e.g., IBD), this reflects an enrichment effect and suggests a genetic overlap between the two traits (16). The generation of Q–Q plots in this study was carried out using the precimed/mixer package^2^ in Python 3.11.

### 2.4 CondFDR/ConjFDR analysis

Within the empirical Bayesian statistical framework, condFDR and conjFDR were introduced by Ole A. A statistical method for identifying shared risk loci between two traits was proposed by Andreassen et al. (17). In comparison to other approaches, this method is capable of identifying loci that do not meet the significance threshold, while maintaining high confidence in the loci detected (17). In condFDR analysis involving two traits, one trait (e.g., alcohol intake) is used as a reference for genetic loci, while the genetic loci associated with the other trait (e.g., IBD) are then screened (18). This method utilizes the association between variants and one trait (e.g., alcohol intake) to reorder the test statistics and recalculate the associations between these variants and the second trait (IBD). The process is bidirectional, and the conjFDR value represents the maximum of the condFDR values derived from the bidirectional analysis. Subsequently, conjFDR analysis is performed to identify genetic loci shared by both traits. The significance level was set at “condFDR ≤0.05.” Detailed analysis procedures for conjFDR are available on the website^3^. The SNPs associated with the identified loci were uploaded to the SNP2Gene module of FUMA^4^ (19) for the identification of annotated genes.

### 2.5 Cross-trait meta-analysis

The MTAG analysis for alcohol and cheese intake and IBD (including CD and UC) was conducted using Python 3.11.5 (11). MTAG, as an alternative statistical method for detecting genetic risk variants across traits, offers the advantage of enhanced statistical power and broader applicability (11). Furthermore, MTAG effectively addresses the issue of sample overlap. The methodology is based on the shared variance–covariance matrix of effect sizes across different traits (11). Upon completion of the MTAG analysis, a GWAS dataset related to both traits was acquired. This dataset was subsequently uploaded to FUMA (19) to identify common genetic risk loci and tissue enrichment results.

## 3 Results

### 3.1 Global and local genetic correlation

In the LDSC analysis, significant positive genetic correlations were identified between alcohol intake and IBD (rg = 0.0835, *p* = 0.0045), as well as with CD (rg = 0.1207, *p* = 2.163e-05), whereas the correlation with UC did not attain significance (rg = 0.0219, *p* = 0.5085) (Table 1). In contrast, a negative genetic correlation was observed between cheese intake and IBD (rg = −0.0929, *p* = 0.0028), as well as with CD (rg = −0.1483, *p* = 5.2972e-06), while the correlation with UC remained non-significant (rg = −0.0112, *p* = 0.7482) (Table 1). These findings were further supported by the GNOVA data (Table 1).

**Table 1:** Genetic correlation of alcohol, cheese intake and IBD (including CD and UC).

Trait1	Trait2	LSDC-Genetic correlation	LSDC-P	GNOVA-Genetic correlation	GNOVA-P
IBD	Alcohol intake	0.0835	0.0045	0.0607	0.0046
CD	Alcohol intake	0.1207	2.163e-05	0.102	1.2958e-06
UC	Alcohol intake	0.0219	0.5085	0.0057	0.7945
IBD	Cheese intake	−0.0929	0.0028	−0.0546	0.0224
CD	Cheese intake	−0.1483	5.2972e-06	−0.1111	5.4474e-06
UC	Cheese intake	−0.0112	0.7482	−0.0232	0.331

IBD, inflammatory bowel disease; CD, Crohn’s disease; UC, ulcerative colitis.

From the local genetic correlation findings using SUPERGNOVA analysis, it was identified that alcohol intake and IBD had positive correlations on chromosomes 16, 10, and 4. In terms of CD, a negative correlation with alcohol intake was detected on chromosome 19, with positive correlations noted on chromosomes 5, 10, and 16. For UC, only a positive correlation on chromosome 16 was identified (Table 2). Cheese intake displayed negative correlations with both IBD and its subtypes (CD and UC) on chromosome 16. Additionally, CD located on chromosome 5 is negatively correlated with cheese intake (Table 2). Comprehensive analytical details are presented in Supplementary Tables S1–S6.

**Table 2:** The results of local genetic correlation between alcohol, cheese intake and IBD and CD.

Trait1	Trait2	chr	Start	End	Genetic correlation	h2_1	h2_2	*p*
IBD	Alcohol intake	16	27,446,054	29,023,966	0.990260824	0.001852106	0.000596502	1.17E-08
IBD	Alcohol intake	10	33,656,119	36,017,592	0.815440581	0.002409804	0.000109213	5.26E-07
IBD	Alcohol intake	4	1,478,711	3,612,028	0.791045237	0.000826533	0.000299523	1.71E-05
CD	Alcohol intake	19	48,697,961	49,485,758	−0.91004321	0.001832887	0.000313803	1.91E-08
CD	Alcohol intake	5	95,822,732	96,867,936	1.123421517	0.002747509	5.98E-05	9.19E-08
CD	Alcohol intake	16	27,446,054	29,023,966	0.996540504	0.002146098	0.000586489	1.50E-07
CD	Alcohol intake	10	33,656,119	36,017,592	0.802529366	0.004476241	0.000109311	2.20E-07
UC	Alcohol intake	16	27,446,054	29,023,966	1.002533886	0.000973262	0.000586455	4.23E-09
IBD	Cheese intake	16	27,446,054	29,023,966	−1.07026065	0.001852106	0.000135665	6.36E-08
CD	Cheese intake	16	27,446,054	29,023,966	−1.074673695	0.002146098	0.000133144	3.93E-07
CD	Cheese intake	5	95,822,732	96,867,936	−0.910343556	0.002747509	0.000109742	2.14E-06
UC	Cheese intake	16	27,446,054	29,023,966	−1.084419189	0.000973262	0.000133136	2.07E-06

h2: represents the observed genetic contribution, the larger the better. P: the statistically significant association is defined to be *p* < 0.05/2353 = 2.12495E-05. IBD, inflammatory bowel disease; CD, Crohn’s disease.

### 3.2 ConjFDR analysis identifies shared genomic loci between two traits

**Figure 2:**
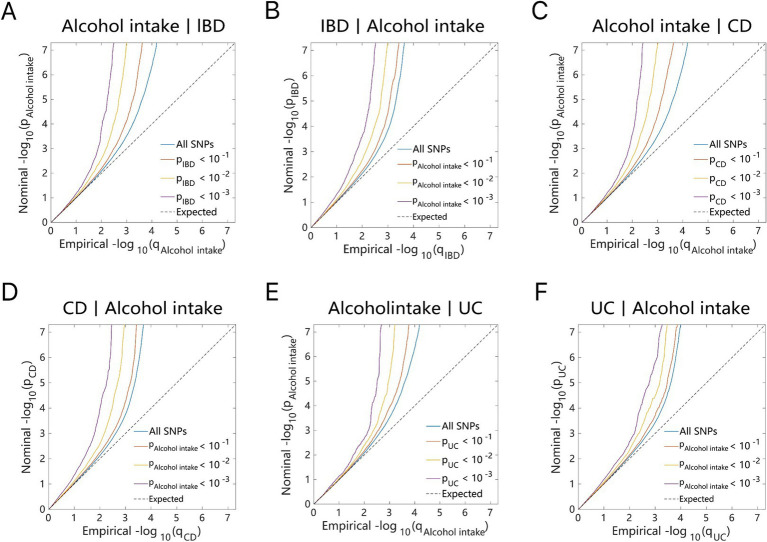
Conditional quantile-quantile plot. The dashed line indicates the expected line under the null hypothesis, and the deflection to the left indicates the degree of pleiotropic enrichment. **(A)** Alcohol intake-IBD. **(B)** IBD-Alcohol intake. **(C)** Alcohol intake-CD. **(D)** CD-Alcohol intake. **(E)** Alcohol intake-UC. **(F)** UC-Alcohol intake. IBD, inflammatory bowel disease; CD, Crohn’s disease; UC, ulcerative colitis.

**Figure 3:**
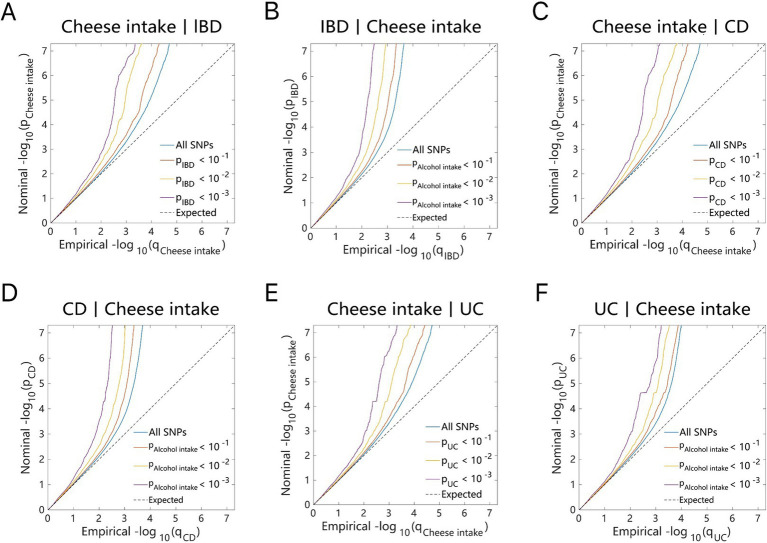
Conditional quantile-quantile plot. The dashed line indicates the expected line under the null hypothesis, and the deflection to the left indicates the degree of pleiotropic enrichment. **(A)** Cheese intake-IBD. **(B)** IBD-Cheese intake. **(C)** Cheese intake-CD. **(D)** CD-Cheese intake. **(E)** Cheese intake-UC. **(F)** UC-Cheese intake. IBD, inflammatory bowel disease; CD, Crohn’s disease; UC, ulcerative colitis.

As illustrated by the Q–Q plots (Figures 2, 3), it was noted that with the decrease in association *p*-values for one trait (e.g., alcohol intake), there is an evident increase in the leftward shift of the association curve for another trait (e.g., IBD). This pattern suggests a robust correlation between these two traits, implying the presence of common genetic risk loci and genetic overlap.

**Figure 4:**
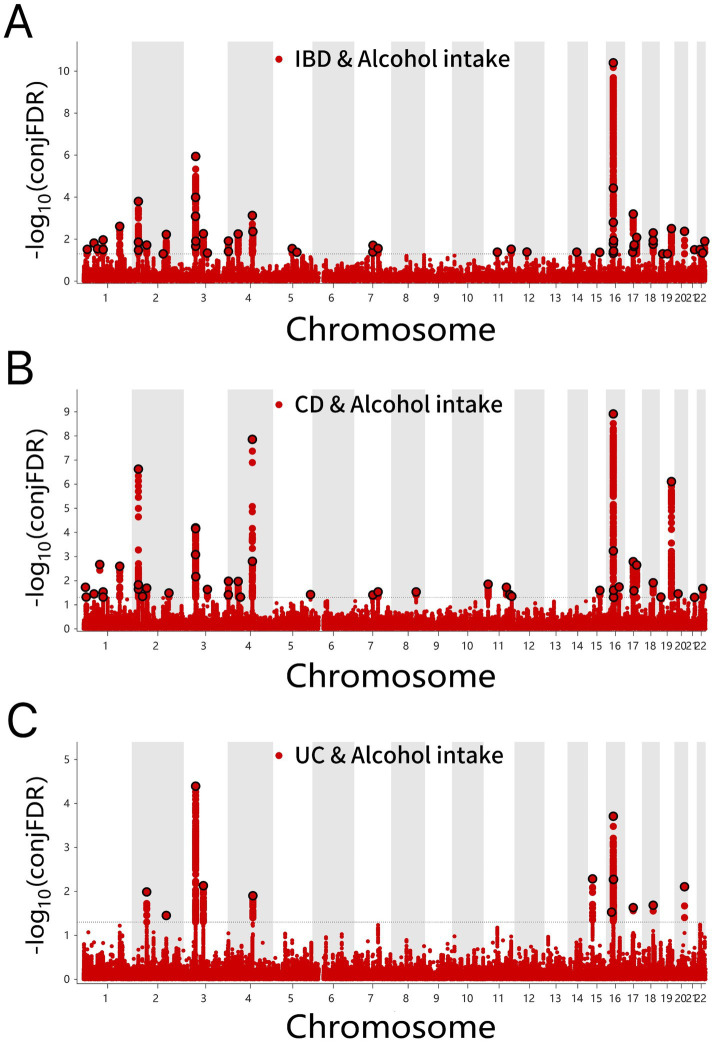
**(A)** ConjFDR Manhattan plot of IBD and alcohol intake. **(B)** ConjFDR Manhattan plot of CD and Alcohol intake. **(C)** ConjFDR Manhattan plot of UC and alcohol intake. The shared risk loci between Alcohol intake and IBD, CD and UC were marked. The statistically significant causality is defined to be conjFDR < 0.05. IBD, inflammatory bowel disease; CD, Crohn’s disease; UC, ulcerative colitis.

Through ConjFDR analysis, high-confidence shared risk loci were identified after processing the overlapping genes for two traits. When conjFDR <0.05, a total of 57 shared risk loci were identified between alcohol intake and IBD, among which 37 genes exhibited consistent directional effects for both traits (Z > 0 for both) (Figure 4A and Supplementary Table S7). For alcohol intake, 42 shared genetic loci were identified in CD and 12 in UC, with 31 and 6 genes showing consistent directional effects, respectively (Figures 4B,C and Supplementary Tables S8, S9).

**Figure 5:**
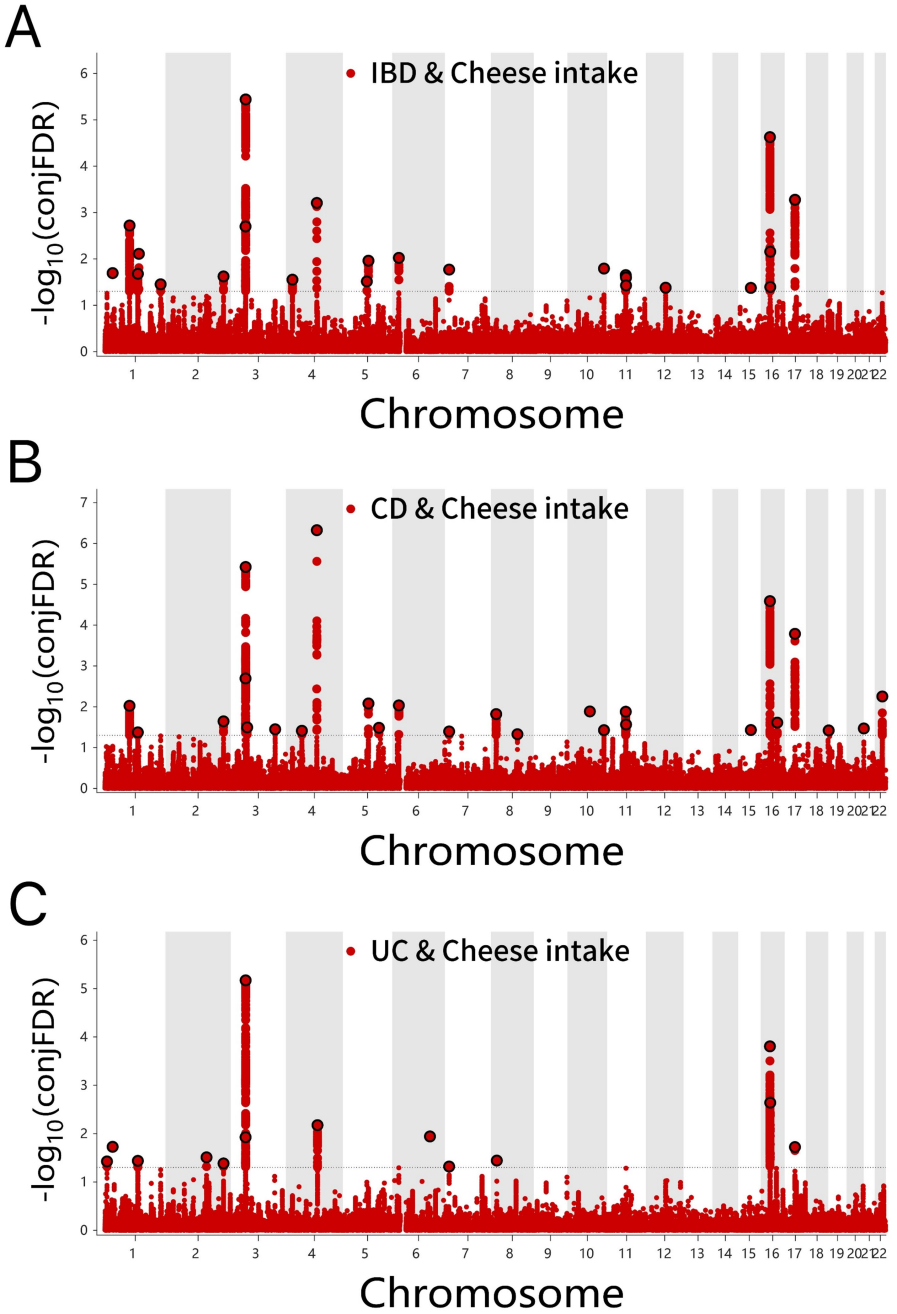
**(A)** ConjFDR Manhattan plot of IBD and cheese intake. **(B)** ConjFDR Manhattan plot of CD and Cheese intake. **(C)** ConjFDR Manhattan plot of UC and Cheese intake. The shared risk loci between Cheese intake and IBD, CD and UC were marked. The statistically significant causality is defined to be conjFDR < 0.05. IBD, inflammatory bowel disease; CD, Crohn’s disease; UC, ulcerative colitis.

Several shared loci were identified for cheese intake in IBD (24), CD (26), and UC (14) conditions (Figures 5A–C and Supplementary Tables S10–S12). Consistent effects of cheese intake were observed for six genes in IBD, eight genes in CD, and six genes in UC, respectively.

### 3.3 MTAG

**Figure 6:**
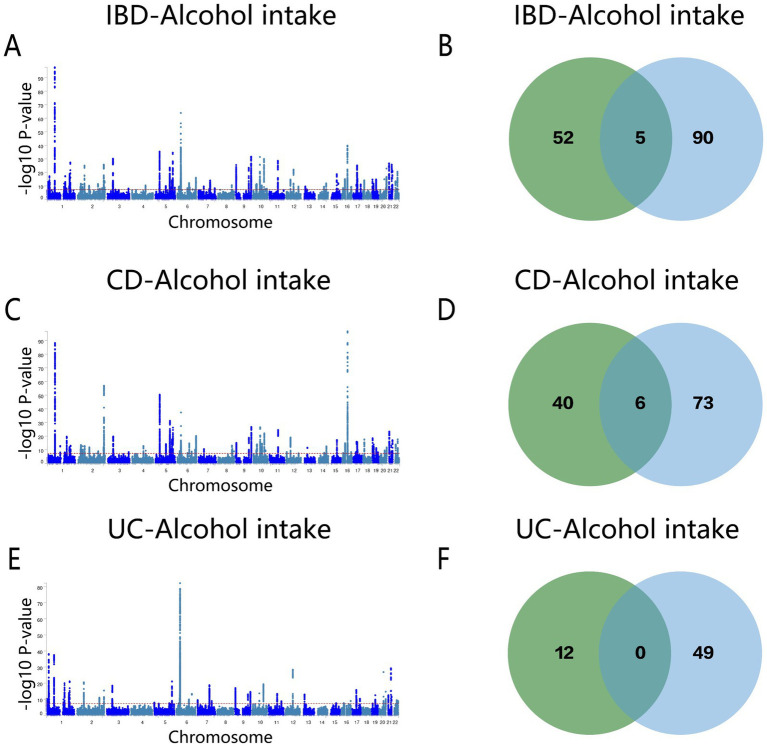
**(A)** Manhattan map of genetic risk loci for IBD and Alcohol intake by MTAG. **(B)** Intersection gene map of IBD and Alcohol intake after conjfdr and MTAG analysis. **(C)** Manhattan map of genetic risk loci for CD and Alcohol intake by MTAG. **(D)** Intersection gene map of CD and Alcohol intake after conjfdr and MTAG analysis. **(E)** Manhattan map of genetic risk loci for UC and alcohol intake by MTAG. **(F)** Intersection gene map of UC and Alcohol intake after conjfdr and MTAG analysis. IBD, inflammatory bowel disease; CD, Crohn’s disease; UC, ulcerative colitis.

Following the MTAG analysis of alcohol intake and IBD, a GWAS dataset relevant to both traits was derived. Subsequent Fuma annotation of these results identified 94 shared risk loci (Figure 6A and Supplementary Table S13), of which five genes (*Y_RNA, DENND1B, GCKR, KPNA*, and *CLN3*) overlapped with the conjFDR analysis results (Figure 6B). For CD, 79 genes were recognized (Figure 6C and Supplementary Table S14), among these, *Y_RNA, DENND1B, GCKR, SLC39A8, CLN3*, and *FUT2* were consistently identified in both conjFDR and MTAG analyses (Figure 6D). The MTAG analysis for UC revealed 49 shared loci (Figure 6E and Supplementary Table S15), although no common genes were identified between the conjFDR and MTAG analyses for this condition (Figure 6F).

**Figure 7:**
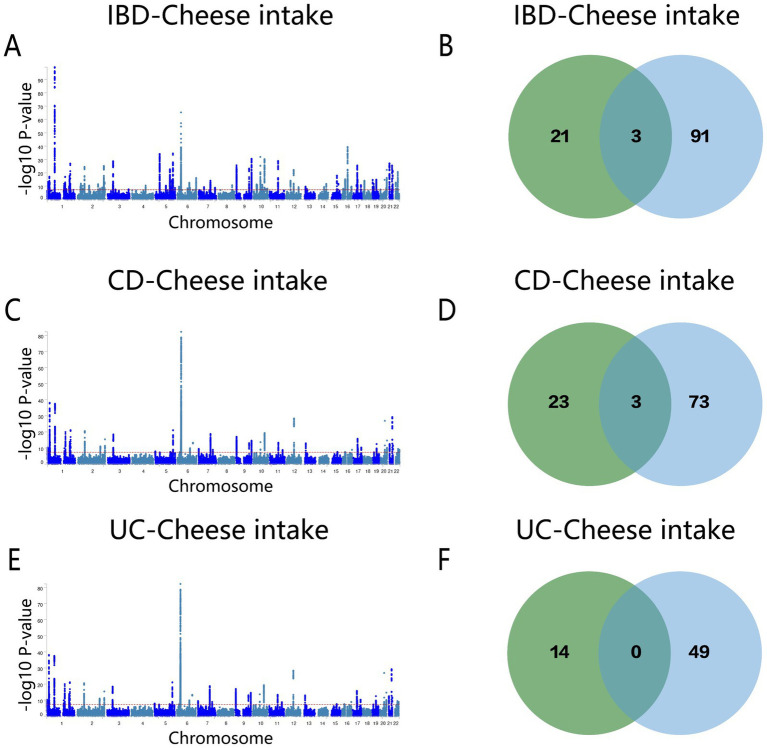
**(A)** Manhattan map of genetic risk loci for IBD and cheese intake by MTAG. **(B)** Intersection gene map of IBD and cheese intake after conjfdr and MTAG analysis. **(C)** Manhattan map of genetic risk loci for CD and cheese intake by MTAG. **(D)** Intersection gene map of CD and Cheese intake after conjfdr and MTAG analysis. **(E)** Manhattan map of genetic risk loci for UC and Cheese intake by MTAG. **(F)** Intersection gene map of UC and Cheese intake after conjfdr and MTAG analysis. IBD, inflammatory bowel disease; CD, Crohn’s disease; UC, ulcerative colitis.

The MTAG results for cheese intake and IBD indicated 94 shared loci (Figure 7A and Supplementary Table S16). *ERAP2, SMAD3*, and *Y_RNA* were pinpointed as overlapping genes in both conjFDR and MTAG analyses (Figure 7B). For CD, 25 shared loci were identified (Figure 7C and Supplementary Table S17), with *SLC39A8, ERAP2*, and *SMAD3* being the overlapping genes (Figure 7D). The MTAG analysis for UC revealed 49 shared loci (Figure 7E and Supplementary Table S18), but no overlapping genes were identified between the two analyses (Figure 7F).

## 4 Discussion

This study conducted a comprehensive genetic analysis of the relationship between alcohol and cheese intake and IBD, including its subtypes. By employing both genome-wide and localized genetic methods, we uncovered important insights into these associations. These findings not only enhance our understanding of IBD pathogenesis but also provide new perspectives on the role of dietary factors in complex diseases.

First, our genome-wide genetic correlation analysis revealed a significant positive association between alcohol consumption and both IBD and CD, while cheese intake showed a negative correlation. These associations align with emerging evidence suggesting that alcohol consumption may exacerbate intestinal inflammation through its effects on the gut microbiome (20). Previous observational studies have reported that alcohol use disorder shares microbial characteristics with those observed in IBD patients (21, 22), and large retrospective studies have shown an increased IBD risk among individuals with alcohol use disorder. This may be attributed to excessive alcohol consumption disrupting gut permeability and increasing bacterial translocation (23). Notably, neither alcohol nor cheese intake showed a significant association with UC. Consistent with our findings, a prospective cohort study investigating the dietary habits of UC patients in remission reported no significant risk of UC relapse with moderate alcohol consumption (24). These findings highlight the differential impacts of dietary factors on IBD subtypes, particularly CD, where stronger associations were observed.

Our study adds a genetic perspective to these observations, minimizing confounding factors and providing more robust evidence for the causal relationship between alcohol intake and IBD. In contrast, cheese consumption, a major source of fermented foods, may protect against IBD by modulating the gut microbiota and reducing markers of systemic inflammation (25). Experimental studies in mice have shown that whey protein from cheese production can enhance mucin synthesis and stimulate beneficial gut microbiota, alleviating colitis symptoms (26). Our findings complement these studies by identifying genetic evidence supporting the protective effects of cheese intake, particularly against CD. However, cheese consumption had no significant impact on UC. This disparity may be linked to the distinct pathological characteristics of these diseases, with CD affecting any part of the gastrointestinal tract in a discontinuous pattern, while UC is restricted to the colonic mucosa (27).

Secondly, local genetic analysis identified chromosome 16 as a key region mediating the effects of alcohol and cheese intake on IBD, including its subtypes. This region is particularly intriguing due to the presence of genes such as *SLC39A8*, *FUT2*, and *ERAP2*, which play roles in immune regulation and gut health (28–30). For example, variations in the *FUT2* gene are associated with alterations in gut microbiota composition, which may influence individual susceptibility to IBD (31). Additionally, the *ERAP2* gene, critical for antigen presentation and immune responses, has variations that could lead to abnormal reactions to gut microbiota, promoting the development of IBD (32). The observed positive and negative correlations of alcohol and cheese intake with IBD, respectively, further support the hypothesis that this genomic region may serve as a nexus for the interaction between diet and genetics in influencing IBD.

Thirdly, our study identified nine shared genes—*Y_RNA*, *DENND1B*, *GCKR*, *KPNA7*, *CLN3*, *SLC39A8*, *FUT2*, *ERAP2*, and *SMAD3*—linking dietary intake and IBD. These findings provide novel insights into the genetic mechanisms underlying these associations. Among these, *Y_RNA*, *DENND1B*, *GCKR*, and *KPNA7*, validated as risk genes (*Z* > 0), likely contribute significantly to the connection between alcohol intake and IBD. For instance, *KPNA7*, a regulator of NF-κB signaling, plays a key role in inflammatory changes in IBD (33). *DENND1B*, associated with various aspects of immune function, may promote IBD through its effects on cell signaling and inflammatory responses (34). Prior GWAS studies have confirmed *DENND1B* as a susceptibility locus for CD (35). Meanwhile, *Y_RNA*, a non-canonical RNA linked to immune signaling and inflammatory diseases, has emerging evidence associating it with alcohol-related immune pathways (36–38). Certain variations in the *GCKR* gene may influence an individual’s metabolism and response to alcohol. Additionally, these variations are associated with susceptibility to IBD, potentially affecting intestinal barrier function (39). The association between *CLN3*, alcohol consumption, and IBD requires further investigation.

For cheese intake and IBD, *SMAD3* stood out as a protective gene for CD and IBD. *SMAD3* is involved in TGF-*β* signaling, and its deletion impairs TGF-β pathway functionality, hindering mucosal healing in CD mouse models. This underscores its potential role in maintaining gut homeostasis (40, 41). *ERAP2* is another significant gene identified in the relationship between cheese intake and IBD. Variations in *ERAP2* may modulate the response to lactic acid bacteria or fatty acids present in cheese, thereby influencing IBD susceptibility and symptoms (42). *SLC39A8* encodes a zinc/manganese transporter protein that participates in various cellular metabolic processes and is closely associated with the pathogenesis of IBD (43). Cheese, as a dairy product rich in manganese and zinc, may indirectly affect IBD through its impact on trace element balance and gut microbiota (44). The identification of these shared loci highlights potential therapeutic targets and provides a genetic foundation for exploring dietary modifications as strategies for the prevention or management of IBD.

This study makes a significant contribution to understanding the genetic basis of dietary influences on IBD. By integrating genome-wide and local genetic approaches with multiple statistical methods, we minimized biases and enhanced the robustness of our findings. However, certain limitations should be acknowledged. First, the potential for linkage disequilibrium cannot be entirely excluded. Second, despite employing rigorous methods to control for confounding factors, residual influences from behavioral, social, and environmental factors may persist. The GWAS data on alcohol intake did not distinguish between fermented alcoholic beverages (e.g., wine and beer) and distilled alcoholic beverages (e.g., spirits). This lack of classification may impact the precision and interpretability of the observed associations between alcohol intake and disease risk. Future studies could focus on developing datasets that differentiate between types of alcoholic beverages to explore their distinct effects on disease more comprehensively. Lastly, our findings are limited to individuals of European ancestry, which restricts the generalizability of the results. Future research should include diverse populations and incorporate functional validation to strengthen the clinical and biological relevance of these findings.

## 5 Conclusion

In summary, our findings provide novel genetic insights into the relationship between alcohol and cheese intake and IBD. The identification of shared loci, particularly those involved in immune regulation and microbiome interactions, offers valuable perspectives on diet-gene interactions in IBD. These results not only advance the current understanding of IBD etiology but also pave the way for personalized dietary recommendations and targeted therapeutic interventions.

## Data Availability

The original contributions presented in the study are included in the article/Supplementary material, further inquiries can be directed to the corresponding author.

## Footnotes

^1^ https://gwas.mrcieu.ac.uk/

^2^ https://github.com/precimed/mixer

^3^ https://github.com/precimed/pleiofdr

^4^ https://fuma.ctglab.nl/
